# Spread of Avian Influenza Viruses by Common Teal (*Anas crecca*) in Europe

**DOI:** 10.1371/journal.pone.0007289

**Published:** 2009-10-05

**Authors:** Camille Lebarbenchon, Frédéric Albespy, Anne-Laure Brochet, Viviane Grandhomme, François Renaud, Hervé Fritz, Andy J. Green, Frédéric Thomas, Sylvie van der Werf, Philippe Aubry, Matthieu Guillemain, Michel Gauthier-Clerc

**Affiliations:** 1 Centre de Recherche de la Tour du Valat, Le Sambuc, Arles, France; 2 GEMI, UMR CNRS/IRD 2724, IRD, Montpellier, France; 3 Unité de Génétique Moléculaire des Virus à ARN, URA3015 CNRS, EA302 Université Paris-Diderot, Institut Pasteur, Paris, France; 4 ONCFS, CNERA-Avifaune migratrice La Tour du Valat, Le Sambuc, Arles, France; 5 ONCFS, Direction des Etudes et de la Recherche, Le Perray-en-Yvelines Cedex, France; 6 Université de Lyon, Université Claude Bernard Lyon 1, CNRS UMR 5558 Biométrie et Biologie Evolutive, Villeurbanne cedex, France; 7 Department of Wetland Ecology, Estación Biológica de Doñana-CSIC, C/Américo Vespucio s/n, Sevilla, Spain; 8 Institut de Recherche en Biologie Végétale, Département de sciences biologiques, Université de Montréal, rue Sherbrooke est Montréal (Québec), Canada; New Mexico State University, United States of America

## Abstract

Since the recent spread of highly pathogenic (HP) H5N1 subtypes, avian influenza virus (AIV) dispersal has become an increasing focus of research. As for any other bird-borne pathogen, dispersal of these viruses is related to local and migratory movements of their hosts. In this study, we investigated potential AIV spread by Common Teal (*Anas crecca*) from the Camargue area, in the South of France, across Europe. Based on bird-ring recoveries, local duck population sizes and prevalence of infection with these viruses, we built an individual-based spatially explicit model describing bird movements, both locally (between wintering areas) and at the flyway scale. We investigated the effects of viral excretion duration and inactivation rate in water by simulating AIV spread with varying values for these two parameters. The results indicate that an efficient AIV dispersal in space is possible only for excretion durations longer than 7 days. Virus inactivation rate in the environment appears as a key parameter in the model because it allows local persistence of AIV over several months, the interval between two migratory periods. Virus persistence in water thus represents an important component of contamination risk as ducks migrate along their flyway. Based on the present modelling exercise, we also argue that HP H5N1 AIV is unlikely to be efficiently spread by Common Teal dispersal only.

## Introduction

Wild birds, especially waterbirds such as Anseriformes (ducks, geese and swans) and Charadriiformes (gulls, terns and waders), are natural hosts for influenza A viruses [1], [2]. As a consequence, avian influenza virus (AIV) dispersal ability is directly linked to host bird dispersal, through migration as well as other kinds of movements (between wintering areas, from roost to foraging sites, etc.). The recent spread of highly pathogenic (HP) H5N1 AIV has raised the question of how, when and where such pathogens could spread via migratory birds [3]–[5]. Despite the few cases of HP AIV reported in healthy wild living ducks [6], [7], it remains highly complicated to predict the spatiotemporal dynamics of the spread of viruses that depend on the movement patterns of their bird host species [5], [8]–[10].

Wild bird migrations have been extensively studied throughout the world [11]. Timing, durations, flyways, stopover areas and other ecological aspects of migration are better understood thanks to a growing number of new tracking techniques. For instance, satellite telemetry and global positioning systems have opened new possibilities to study movements of wild animals. However, the weight and cost of such data loggers limit their use to the largest species and to small sample sizes [12]. Ring recovery datasets (capture-mark-recapture method) do not have such limitations, and thus remain the basis for the large-scale study of wild bird movements (e.g. [13]).

Common Teal (*Anas crecca*) is among the most abundant duck species in Europe [14], [15]. After breeding, in Siberia and Northern Europe, this species undertakes fall migration in August-September to spend the winter in Western Europe, until spring migration starts again in February from southern wintering grounds [14], [16]. Both migratory and wintering movements of Teal have been extensively studied [17], [18]. This has especially been the case from the South of France, in the Camargue area, where 59 087 of such ducks were ringed between 1952 and 1978 [19]–[22]. Prevalences of AIV infection are particularly high (from 3.6% to 12.9%) in both breeding and wintering areas [23]–[25], suggesting an important role for Common Teal in the ecology and epidemiology of these viruses in Europe.

In this study, we investigated AIV dispersal by Common Teal, from the Camargue area, by developing a computer model reproducing migratory and local movements (i.e. during the wintering period) of ducks. The aims of this study were to: (i) Build an individual-based spatially explicit model which describes duck movements in space and time. Based on the ringing dataset and duck abundance in the Camargue, we reproduced Common Teal movements in Europe, from September (beginning of the wintering period) to May (end of spring migration). (ii) Spatially represent the maximal AIV spread distance by this species (under the assumption that infected ducks were not subjected to behavioral modifications reducing flight abilities). We integrated monthly AIV prevalence data recorded in the Camargue and simulated virus spread according to a large set of hypothetical viral excretion duration values (from 2 to 30 days, [26]–[28]). (iii) Investigate how virus persistence in water could affect AIV spread. We addressed this point by testing a large set of hypothetical values for virus inactivation rate in water (from 7 to 207 days, [29], [30]). (iv) Simulate HP H5N1 AIV dispersal by Common Teal, especially during spring migration, by integrating empirical measures of HP H5N1 excretion duration and virus inactivation rate in water provided by experimental studies [31], [32].

## Results

### Common Teal simulated movements

**Figure 1:**
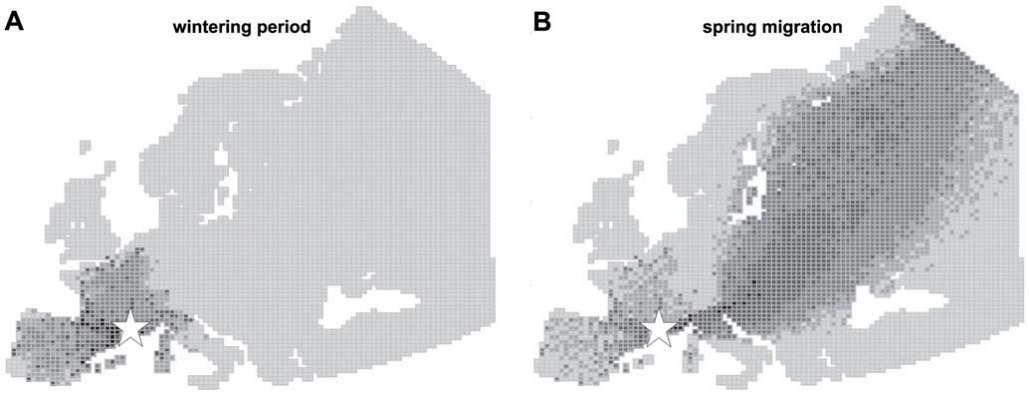
Simulations of Common Teal movements in Europe, from the Camargue, South of France (white star). A: Wintering period (September to January). B: Spring migration (February to May). Grey scale represents the number of simulated bird halts in each mesh: light grey: no halt; intermediate greys: 1–5, 6–50, 51–500; black: more than 501 halts.

From September to January, Common Teal movements away from the Camargue were limited to wintering sites in Western Europe, mainly on the French Atlantic coast, the Spanish East Mediterranean coast and the Pyrenees (Figure 1A). From February onwards, ducks then moved mainly along a South West–North East axis, from the Camargue to Eastern Europe and Scandinavia (Figure 1B). Common Teal left the Camargue in February and March to undertake spring migration, and moved all the way to breeding sites where they arrive until the end of May. Late local movements also occurred (mostly in France and Spain), mainly in February, but to a lesser extent than during winter.

### Circulation of AIV in the Camargue

Fifty-five birds infected by AIV were detected among the 799 Common Teal sampled between September 2007 and February 2008 (6.9% of sampled ducks). This prevalence was higher than those previously reported in the Camargue [23], [33], but consistent with other European studies [24], [25]. We did not detect HP H5N1 AIV but seven LP (Low Pathogenic) H5 AIV were found.

We found a clear pattern of circulation, consistent with previous studies [23], [33], with significantly higher prevalence rates in September (GLM; odds ration = 2.79; confidence interval = [0.22–35.56]; P<0.01). Such seasonal variation with higher prevalence recorded in early fall has also been reported elsewhere in Europe [24]. In the Camargue, high prevalence in early fall may be explained by the arrival of large numbers of young and possibly immunologically naïve birds. These birds may either bring AIV or become infected with locally circulating AIV subtypes. Hence, specific immune responses could develop during the wintering period, explaining the decreasing rate of infected ducks observed each year from September to February (Figure S1; [23], [33]).

### Effect of high and constant prevalence of infected ducks

To avoid a potential bias linked to underestimated prevalence, we also performed simulations with a prevalence of infected ducks equal, each month, to 15%. This value represents the highest infection rate recorded in the Camargue (Figure S1; [23], [33]) and a higher prevalence than those recorded in this species in Northern Europe [24]. In our simulations, such a high and constant prevalence rate induced higher quantities of AIV in each halt mesh (i.e. each stopover site–see movement model section for details), but did not directly affect AIV spread in space and time (Figure S2).

### Effect of viral excretion duration on AIV dispersal

**Figure 2:**
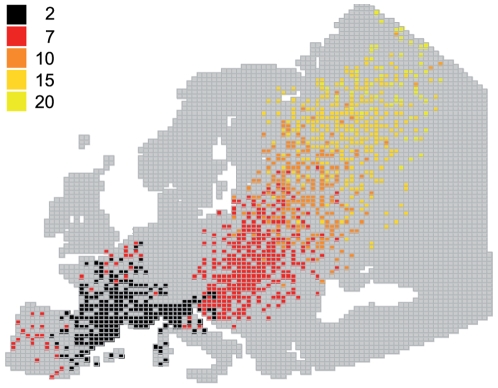
Effect of viral excretion duration on avian influenza virus dispersal. Colours represent different hypothetical values of viral excretion duration (in days).

In this study, we assumed that infected ducks were not subjected to physiological and behavioral modifications reducing movement abilities. Under this scenario, viral excretion duration was likely to be the key parameter for long distance spread of AIV. Simulation results indeed showed clearly that long AIV excretion duration enhanced dispersal efficiency in space (Figure 2). This result however only held true during spring migration, when ducks moved from the Camargue to their breeding sites (Figure 1B), and not during the wintering period. During spring migration, excretion durations less than 15 days were not sufficient to spread AIV circulating in the Camargue directly to Northern Europe and Scandinavia (Figure 2). Viral excretion duration did not have an important effect on AIV dispersal during winter because Common Teal moved only locally, between wintering sites in western Europe (Figure 1A).

### Virus inactivation rate in water

**Figure 3:**
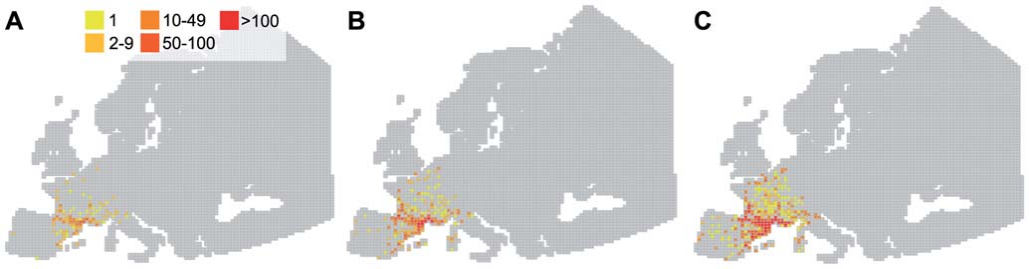
Effect of three virus inactivation rates in water on AIV dispersal during the wintering period (September to January). Hypothetical virus inactivation rates used: A: 7 days; B: 28 days; C: 207 days. Colours represent the number of infectious virus units, on January 31.

**Figure 4:**
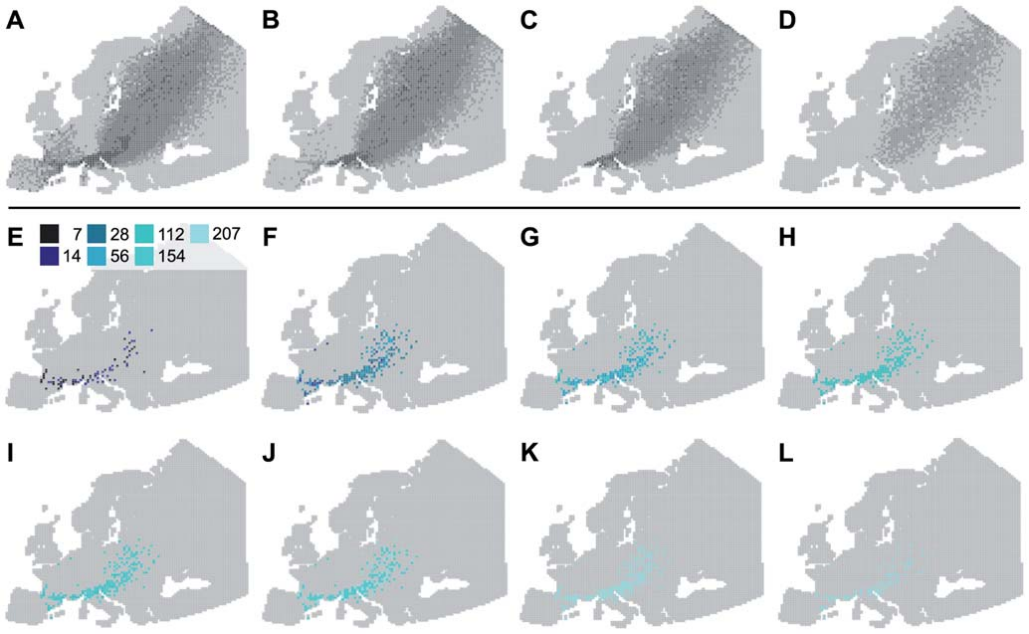
Importance of virus inactivation rates in water during spring migration. A–D: Simulations of Common Teal movements in Europe from February (A) to May (D). The grey scale represents the number of bird halts simulated for each mesh: light grey: no halt; intermediate greys: 1–5, 6–50, 51–500; black: more than 500 halts. E–L: Persistence of AIV in water from February (E) to September (L). Colours represent different hypothetical values of virus inactivation rate. In these simulations, viral excretion duration was fixed at 7 days.

Because of the predominance of local movements of ducks (Figure 1A), virus inactivation rates did not have an important effect on the distance of AIV spread during the wintering period. Locally, low virus inactivation rates increased the quantity of AIV in circulation. Viruses may indeed regularly infect ducks within wintering areas, but also at stopovers between wintering sites (Figure 3). During spring migration, infected ducks leaving the Camargue excreted AIV all along their migratory flyway (Figure 4A–4H). The virus inactivation rate thus has a potential effect on the contamination of non-infected incoming ducks, at an infected stopover site. Finally, after spring migration, AIV with low virus inactivation rates may persist locally during summer (Figure 4I–4L) and potentially infect ducks during fall migration (in August and September).

### HP H5N1 AIV dispersal by Common Teal

**Figure 5:**
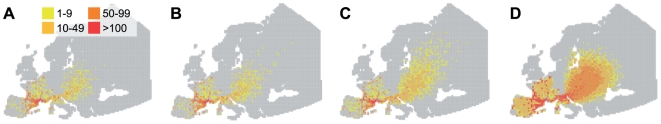
HP H5N1 AIV dispersal by Common Teal in Europe, from the Camargue, from September to May. A. without stopover contamination; with stopover contamination risks: B: low (bird contamination happens when one infectious virus unit is present per 100 000 m^2^ in a given mesh). C: medium (bird contamination happens when one infectious virus unit is present per 1 km^2^ in a given mesh), D: high (bird contamination happens when one infectious virus unit is present in a given mesh). Colours represent the number of infectious virus unit in each mesh.

We simulated HP H5N1 AIV dispersal from the Camargue (Figure 5), according to viral excretion duration recently measured for this species in Europe (6 days [31]) and virus inactivation rate experimentally estimated in water (20 days; [32]). From September to January, HP H5N1 AIV spread locally to other West-European wintering sites. This result directly depended on duck movements during this period (Figure 1A) and short viral excretion duration of this virus (less than 7 days, Figure 2). Large-scale spread of HP H5N1 AIV from the Camargue was thus possible only during spring migration, from February onwards. Because of the short viral excretion duration, however, dispersal efficiency of HP H5N1 AIV was limited, and only a small amount of the viruses or none at all, spread directly from the Camargue to breeding sites in Northern Europe and Scandinavia (Figure 4A).

In these simulations we also investigated three hypothetical contamination scenarios at stopover sites on migrations, as a function of the number of infectious virus units present in halt meshes. We assumed that when a simulated bird left the Camargue in an infected condition, it left an infectious virus unit in each halt mesh it used on its way. This infectious virus unit persisted according to the virus inactivation rate in water. For simplification we did not considered the infective dose needed to initiate an infection but considered that an infectious virus unit always led to infection in a non-infected incoming duck. This assumption overestimated the infection risk at stopover sites.

When this stopover contamination risk was low (Figure 5B), HP H5N1 AIV did not spread over longer distances (except for few simulated birds) than when no stopover contamination was considered (Figure 5A). When considering a medium contamination risk, a small amount of viruses reached the Northern European and Scandinavian breeding sites, through Common Teal migration (Figure 5C). Finally, when high contamination risk was simulated, a more important number of HP H5N1 AIV appeared in each infected mesh, during spring migration but also during the wintering period (Figure 5D). Under this scenario however, the virus did not spread over longer distances than when no, low or medium stopover contamination was considered.

## Discussion

The results provided by simulations of duck movements through our individual-based spatially explicit model were in accordance with studies performed on the ecology of this species in Europe [14], [17], [18], suggesting that model building and parametrization were appropriate, thus adequately mimicking real world situations. Because of the old time series of our ringing data (1952–1978), it is possible that there have been some modifications in Common Teal migratory routes owing to the effects of global change. Numerous studies indeed have investigated the effect of climate change or habitat loss due to human activities, mainly on temporal trends of bird migrations (e.g. [34]–[36]). For Common Teal, climate warming may have important consequences for the distribution of this species in Europe, as more and more birds may become able to remain in northern areas close to their breeding grounds [37]. It is has also been shown that agricultural practices can be responsible for population decline, at the local scale, in European wintering areas [38]. In our study site however, Common Teal population size did not undergo significant reduction since the 1970s [39], suggesting that it still represents a high-risk area for AIV circulation and dispersal during wintering period and spring migration.

The first important aspect highlighted in this study is the need to consider AIV dispersion through wild bird movements according to the period of the year and their biological cycle. For Common Teal, patterns of virus spread are not the same during the wintering period and during spring migration. During winter, local ecological factors such as food availability, hunting or climatic conditions induce local duck movements [19], [40], [41]. From September to January, Common Teal thus move locally, in a non-oriented manner, suggesting a high turnover between wintering sites [42]. During the wintering period, viral excretion duration and inactivation rate in water, as well as local prevalence of AIV infection, influence the quantity of viruses in circulation between wintering sites but have no effect on dispersal distances. From February onwards, Common Teal undertake their migration to North European breeding sites [20]. Duck presence is mainly restricted to geographical areas situated along the migratory flyway, with highest densities midway along the flyway. During spring migration, under the assumption that ducks cannot get infected at stopover sites (i.e. during a halt in an infected mesh), dispersal efficiency of viruses is thus directly dependent on viral excretion duration.

Experimental studies performed on captive-reared ducks often report short viral excretion duration, sometimes with important variability between viral subtypes, bird species and individuals [26], [27], [31], [43], [44]. Our results clearly indicate that AIV with short excretion duration (<7 days) are unlikely to be spread over long distances by wild Common Teal, even during migratory periods, because most of these ducks do not move from wintering sites to breeding areas fast enough. This result thus underlines the necessity to carry out more research on the viral excretion patterns in ducks infected with LP AIV, in the wild. Latorre-Margalef et al. [28] recently reported short duration of infection and virus shedding in wild Mallards (less than 8.3 days on average). They also report that LP AIV infection did not affect speed or distance of subsequent migration. These results suggest that viral excretion duration is unlikely to be the main factor responsible for long distance spread of AIV by wild migratory birds.

In the present model, we assumed that simulated ducks did not get infected during their movements (except for HP H5N1 simulations). Integration of stopover contamination risk requires a precise knowledge of local AIV circulation (prevalence of infection), but also of ecosystem functioning (bird species, population density, etc.), virus inactivation rate in the environment [45], and duck immunity in response to previous infections. The results of our simulations thus represent a simplified picture of real AIV dispersal in the wild. In order to answer more specific questions, our model will undoubtedly need to be refined in the light of future knowledge on the ecology of these viruses, especially in locations used as stopovers during duck migrations.

This study underlines the key role of virus inactivation rate in water, not only at a local scale, but also as an important component of AIV spread over long distances and periods. For many years, water-borne transmission has been suspected to be an important component of AIV epidemiology in wild as well as in domestic ducks [29], [30], [32], [46]–[53]. From September to January, AIV distance spread is not directly linked to virus persistence in water because a large proportion of infected ducks move locally, infecting stopovers and wintering areas regularly. Thus, the lower the virus inactivation rate, the more AIV circulate between wintering sites, due to an important number of infected stopover sites. However, during spring migration, AIV persistence in water is likely to be an important component of contamination along migratory routes. From February to May, a low rate of virus inactivation directly enhances contamination risk at stopover during migration. From June onwards, viruses with an extreme level of persistence in water (more than 154 days) are also likely to infect ducks when they undertake fall migration, in August. Even if lower levels of persistence are considered, viruses may persist locally through local epidemiological cycles by infecting resident (non-migratory) or local breeding birds, thus enhancing AIV infection risk during fall migrations. These aspects show that precise knowledge of local ecosystem functioning is critical in order to assess the period and location of AIV introduction and circulation in wild ducks. For instance, bird species presence and their migratory status (i.e. migrant vs resident), level of AIV circulation and inter specific differences, and abiotic characteristics of aquatic ecosystems are of primary importance to understand how and when these viruses naturally spread from one place to another.

Furthermore, the local prevalence of AIV infection did not have an effect on the distance of virus dispersal outside our study site. In our simulations, high and constant prevalence of infection in Common Teal (15%) over time led to higher quantities of AIV at infected stopover sites and thus, are likely to increase stopover contamination risk during duck migrations. However, such high and constant levels of AIV prevalence of infection before spring migration have never been observed in the Camargue [23], [33]. Before ducks undertake spring migration, AIV circulation is typically low, probably because of an important proportion of immunized ducks. When ducks migrate from wintering sites a strong selection may thus occur and a low diversity of AIV subtypes is likely to be exported to breeding areas. Such a scenario may explain the low genetic diversity of virus strains sampled in the same place or during the same year [54] and could be similar to a source-sink ecological model identified for human influenza A viruses [55].

The spread of the HP H5N1 from Asia to Europe and Africa has been the subject of an intense debate focusing of the role of wild migratory birds (e.g. [3]–[5], [10], [56]–[58]). Because of the short excretion duration of this virus in Common Teal [31], HP H5N1 AIV in Europe is unlikely to be spread efficiently, over long distances, by this species. Moreover, like other AIV subtypes, long distance dispersal is only possible when birds undertake migrations. For HP H5N1, we considered three scenarios of contamination at migratory stopover sites. With a low risk of infection, the observed dispersal pattern was nearly the same as with no stopover contamination. In this scenario we considered that contamination occurs when one infectious virus unit was present per 100 000 m^2^. Increasing contamination risk did not affect the efficiency with which HP H5N1 spread. In our simulations, high stopover contamination risk corresponded to a 100% probability of infection if one infectious virus unit was present per 2 500 km^2^, which is very unlikely from a biological point of view.

Due to the rapid inactivation rate of HP H5N1 in water [32], stopover contamination in infected natural environments does not readily favour viral spread over long distances. During the duck wintering period, HP H5N1 can spread between wintering sites, at a small geographical scale. This result is supported by the cold spell recorded in February 2006 in Eastern Europe, which has been held responsible for the HP H5N1 outbreaks in wild birds in many countries in the European Union [4], [5]. Although this virus may be endemic in domestic and potentially wild birds in China [59], to date HP H5N1 does not continuously circulate in wild duck populations in Europe and Africa. This suggests that HP H5N1 cannot persist in natural ecosystems without regular reintroductions from domestic birds. For large-scale dispersal, recurrent infections along migratory flyways are thus necessary to favour HP H5N1 spread. Domestic birds may act as a source of viruses and thus contribute to sporadic long-distance spread of this virus, through infection of wild birds and subsequent migratory movements. Such a scenario may explain why HP H5H1 is regularly found in wild birds in Asia, where regular outbreaks occur in domestic birds (e.g. [6], [59]), whereas in Europe and Africa it has been detected less often in both wild (e.g. [23], [60]–[64]) and domestic birds [65].

In this study, we assumed that infected wild ducks were not subject to behavioural modification of their movement abilities, in order to measure the maximal AIV spread. To date, physiological and behavioural effects of AIV infections in wild birds have been little studied in nature. However, van Gils et al. [66] recently reported impaired foraging and migration efficiencies in Bewick's Swans (*Cygnus columbianus bewickii*) infected with LP H6 AIV. Latorre-Margalef et al. [28] also showed that body mass was significantly lower in infected wild Mallards (*Anas platyrhynchos*) than in uninfected ones, and that the amount of virus shed by infected juveniles was negatively correlated with body mass. These studies suggest that host physiology and behaviour may be affected by LP AIV viruses in subtle ways not previously envisaged, but they also raise the question of potential subtype and species related specificity linked with AIV dispersal in the wild.

## Materials and Methods

### Ringing and population data

Long-term population studies of Common Teal have been performed in the Camargue (Southern France) between January 1952 and February 1978. In total, 59 087 ducks were ringed (at la “Tour du Valat”, 43°30′N, 4°40′E) among which 9 279 were recovered in Europe, mostly through hunting. In this study, we focused on migratory and wintering movements of Common Teal from this area. We considered only intra-annual ring recoveries (i.e. recovered the same season as the ringing event occurred) to avoid potential biases linked with dispersal from other wintering areas. Indeed, a bird ringed in the Camargue during a given winter may not necessarily return to the Camargue the following winter [22]. Ring recovery data from successive years (i.e. during and after the second winter) may thus not reflect bird movements from our study area, and were not taken into account. In summary, among the 59 087 Common Teal, we used the recovery information from 3 259 individuals, all recoveries being between the ringing date of the bird (92% were ringed between September and February) and the end of August of the same year (Figure S3).

The proportion of Common Teal leaving the Camargue each month was determined based on ring recoveries. For a given month, two types of ring recoveries where distinguished: (i) recoveries performed less than 100 kilometres (km) from the ringing place, assumed to represent birds staying in the Camargue, and (ii) recoveries performed outside this area, corresponding to birds leaving the Camargue. For the later recoveries, we defined whether these flights were local (i.e. between wintering sites) or migratory. According to the biology of this species we considered that all flights more than 100 km from the ringing place, realized between September and January, corresponded to movements between wintering sites [67]. From February to May we considered that Common Teal could either move between the Camargue and another wintering site, or undertake migration from the Camargue to breeding areas [19]–[22]. In another study also based on ring recoveries [42] we found that, in February and March, Common Teal tended to move towards a single direction corresponding to their most likely migration route [20], [21]: from the Camargue to the North East. For simplification, and in order to estimate the proportion of local versus migratory movements during this period (February to May), we arbitrarily drew a line passing by the Tour du Valat, and oriented from the North West to the South East. We then considered that ring recoveries recorded on the southwestern side of the line corresponded to birds moving from the Camargue to another wintering site, and that those recorded Northeastwards corresponded to birds on migration. Finally, from June to August we considered that all ring recoveries corresponded to birds at their breeding sites or undertaking fall migration. These ring recoveries were used to determine mesh preference in northern Europe (see movement model).

Mean abundance of Common Teal in the Camargue was computed between 1964 and 1995 (Figure S4; see [23] or [68] for bird census method). Based on this information and on the monthly proportions of birds staying in or flying away from the Camargue described above, it was then possible to estimate the number of birds staying in the ringing area (the Camargue), moving between wintering sites, and undertaking migration.

Because we used only intra annual ring recoveries, only limited information was available for the September period. We thus considered that September was similar to October in terms of proportion of birds staying in or leaving the Camargue. From a biological point of view this assumption is realistic because both September and October correspond to a migratory period for this species, for which the Camargue is considered to be a stopover as well as a wintering site [20], [68].

### Movement model

An individual-based spatially explicit model was developed to describe bird movements. Space was divided into squared meshes of 50×50 km (projection system of Lambert Azimuthal Equal Area ETRS89, [69]). As described above, we considered two types of bird movements outside the Camargue: (i) migratory flights and (ii) movements between wintering sites. We thus considered two movement rules in this model (migratory and between wintering sites), both being based on an equation describing the use (U) of a mesh (x) [70]: Usage (x) = f ( Accessibility (x), Preference (x) ). Accessibility (A) was defined by an area corresponding to a flight distance and direction. This area contains several meshes that were accessible to a simulated bird. Each day, among these accessible meshes, a simulated bird selected a halt mesh (i.e. stopover site), for instance to rest and feed), as a function of its preference (P). Preference was calculated as the probability as indicated by the number of ring recoveries contained in a given mesh in relation with the total number of ring recoveries of the accessible area recorded all year long. Meshes containing the higher number of ring recoveries were thus considered to be the most attractive ones.

#### (i) Migratory movements rule

Flight direction was determined according to ringing recoveries and previous studies on migration of Common Teal [17], [18], [21]. When a bird left the Camargue as a migrant, flight direction was picked from a uniform distribution between 60° and 100° azimuth (North defined at 0°). When a simulated bird crossed a longitude up to 13° East, the direction was then picked from a uniform distribution between 0° and 90°. This change was set up in order to take into account the Alps as a natural barrier to migratory flights of Common Teal [21]. We then randomly assigned a flight distance from a uniform distribution between 100 and 300 km, according to previous studies performed in this species [42], [71] and on other Anatidae [7], [72]. For each selected direction and flight distance, an interval was randomly determined (±20° and ±50 km respectively). These intervals defined the accessible area (A) in which the simulated bird used a preferred mesh (P). Once they had undertaken migration, simulated birds moved every day (from a mesh to another) until they reached their breeding site, considered in this study as being 63° North or more [14].

#### (ii) Movements between wintering sites

Only meshes with at least one ring recovery during the wintering period (September to January) were selected. We assumed that each simulated bird leaving the Camargue had a well defined destination mesh, in another wintering site. Two scenarios were thus considered: (i) the destination mesh was situated less than 300 km away from the Camargue and the simulated bird was able to reach it directly (i.e. in one single day) or (ii) the destination mesh was situated more than 300 km away from the Camargue and at least one intermediate mesh should be defined (corresponding to a stopover for the bird). In the latter case, the intermediate mesh was determined as for migratory movements. We randomly chose a flight distance in a uniform distribution between 100 and 300 km with an interval of ±50 km. An interval of ±20° around the flight direction (flight direction being the azimuth from the present bird location to the destination mesh) was also determined. In the same manner as for migratory movement, an accessible (A) area was thus defined in which a preferred (P) mesh was determined. The simulated bird movement stopped when the individual reached its destination mesh.

### Avian influenza viruses

AIV dispersal by Common Teal was included in the modeling exercise according to three parameters: (i) monthly prevalence of AIV infection in this duck species in the Camargue, (ii) viral excretion duration and (iii) virus inactivation rate in water.

#### (i) AIV dataset

We sampled 799 Common Teal from September 2007 to February 2008. Freshly killed birds were sampled in seven private hunting marshes (85% of our sampling). Live birds were also caught daily with funnel traps placed at the periphery of wintering marshes in the private natural reserve of “La Tour du Valat”. Cloacal swabs were performed to collect fecal samples and birds were marked with a steel ring before being released.

Cloacal swabs were collected using the Viral Pack kit (Biomedics, S.L.) and kept at −80°C until RNA extraction was performed. Automatic RNA extraction was performed using the BioRobot Mdx workstation and QIAamp Virus BioRobot MDX kit (QIAGEN GmbH) according to the manufacturer's instructions. The presence of influenza viruses was detected by quantitative real-time polymerase chain reaction (q-RT-PCR) targeting the Matrix gene segment, using a LightCycler 480 (Roche). Amplification was performed on 2.5 µL RNA with SuperScript III Platinum One Step Quantitative RT-PCR System (Invitrogen) in the presence of oligonucleotides (0.5 µM) M/52/+ 5′-CTT CTA ACC GAG GTC GAA ACG-3′ and M/253/− 5′-AGG GCA TTT TGG ACA AAK CGT CTA-3′ [73] and a probe M probe/82/+ 5′-[FAM]-CCT CAA AGC CGA GAT CGC GCA-[BHQ1]-3′, using the following cycling conditions: 15 min at 45°C, 3 min at 95°C, then 10 s at 95°C, 10 s at 55°c and 20 s at 72°C repeated fifty times and finally 30 s at 40°C. Positive samples were tested for highly pathogenic HP H5N1 AIV using a q-RT-PCR technique and molecular sequencing of the hemagglutinin cleavage site.

Logistic regressions by General Linear Models (GLM; binomial; R software version 2.9.1) were fitted to the data to investigate the effect of the sampling period in infection status.

#### (ii) Simulation of AIV dispersal

When a simulated bird left the Camargue, its probability of being infected was assigned as the prevalence of AIV infection recorded during the month of its movement. To avoid potential bias linked with underestimated prevalences, we also performed simulations with a prevalence of infected birds equal each month to 15%. This value represents the highest infection rate recorded at our study site since we initiated AIV survey in wild birds in 2005 (see results section and [23], [33]).

For viral excretion duration and virus inactivation rate in water, we investigated a large range of hypothetical values corresponding to possible biologically extreme cases. The following values were tested: viral excretion duration: 2, 7, 10, 15, 20, 25 and 30 days [26]–[28]; and virus inactivation rate in water: 7, 14, 28, 56, 112, 154 and 207 days [29], [30]. In natural conditions, AIV inactivation (or loss of infectivity) in water over time decreases at a log-linear rate, and a high variety of responses have been described according subtypes and environmental conditions [50]. For simplification, we defined virus inactivation rate as the period during which a virus remain infectious in the environment, considering that infectivity is constant through time.

In our model, we assumed that simulated birds did not get infected during their movements. When a simulated bird left the Camargue being in a healthy state, it moved to its destination mesh (breeding area or other wintering site) without contamination at stopovers. This assumption is the main limitation in our model and led to the further assumptions that AIV circulation did not previously occur in halt meshes, or that infected birds became immunized and cannot be re-infected during their movements. Although the stopover contamination risk was not possible to predict in our general AIV model (because of too many hypothetical scenarios regarding excretion duration period and virus inactivation rates in water, in halt meshes), we investigated the case of HP H5N1 dispersal, using fixed values of viral excretion duration and virus inactivation rate in water, corresponding to those provided in the literature.

### Highly pathogenic H5N1 simulations

We investigated dispersal of HP H5N1 AIV by Common Teal by considering a hypothetical circulation in the Camargue and integrating recent data concerning viral excretion duration and virus inactivation rate in water. For viral excretion duration we used the maximal excretion length (6 days) recently provided by Keawcharoen et al., on European Common Teal [31]. For HP H5N1 AIV inactivation rate in water, we used the average time (20 days) required to reduce the initial virus concentration by 90%, at 17°C (for A/Whooper Swan/Mongolia/244/05(H5N1) (Mongolia/05) and A/Duck Meat/Anyang/01 (H5N1) (Anyang/01), c.f. [32]). Simulations of bird movements to study HP H5N1 dispersal were performed as described above.

Because excretion duration and inactivation rates in water were fixed for HP H5N1, we were able to investigate a hypothetical contamination risk at stopovers. We assumed that when a simulated bird left the Camargue in an infected condition, it left an infectious virus unit in each halt mesh it used on its way. This infectious virus unit persisted according to the virus inactivation rate in water. We thus considered for each mesh a stopover contamination risk depending on the number of infectious virus units in each mesh (i.e. the number of infected birds which had stopped in a given mesh before and the virus inactivation rate). We considered that an infectious virus unit always led to infection in a non-infected bird.

We defined the stopover contamination probability as a linear function of the number of infectious virus units present in each mesh at the arrival date of a simulated bird. The more infected birds use a given mesh, the higher the density of infectious virus units and thus stopover contamination probability is higher. We tested 3 hypothetical scenarios, corresponding to 3 densities of infectious virus unit thresholds in halt meshes: (i) low contamination risk: a simulated healthy bird gets infected if the density of infectious virus units in the halt mesh is equal to 1 per 100 000 m^2^ (i.e. 25 000 infectious virus units per mesh), (ii) medium contamination risk (1 per km^2^, i.e. 2 500 infectious virus units per mesh) and (iii) a high, or extreme contamination risk (1 per 2 500 km^2^, i.e. contamination for even 1 infectious virus unit per mesh).

## Supporting Information

Figure S1 Avian influenza virus prevalence in Common Teal in the Camargue, during winter 2007–2008 (triangles represent 95% confidence interval).
(0.02 MB PDF)

Figure S2 Effect of local prevalence of infection on AIV dispersal during the wintering period (A, B) and spring migration (C, D). Maps represent AIV circulation with prevalence measured during the 2007–2008 season (A, C) and with a hypothetical constant monthly prevalence of 15% (B, D). Colours represent the maximum number of infectious virus units per mesh: intermediate blue: 1, 2 to 10, 11 to 49, 50 to 99, 100 to 200; black: more than 200 infectious virus units.
(7.57 MB EPS)

Figure S3 Common Teal ring recoveries (September to May) recorded in Europe between January 1952 and February 1978. Green scale color represents the number of ring recoveries recorded on each mesh: light green: 1 to 4; intermediate green: 5 to 29; dark green: more than 30 ring recoveries. The white star represents the geographic location of the Camargue.
(2.17 MB EPS)

Figure S4 Mean abundance (and standard deviation) of Common Teal in the Camargue, computed between 1964 and 1995.
(0.02 MB PDF)
