# Supplementary figures and images for: Spread of Avian Influenza Viruses by Common Teal (Anas crecca) in Europe
Source: PLoS One. 2009 Oct 5;4(10):e7289. doi: 10.1371/journal.pone.0007289 (PMC2750755; doi:10.1371/journal.pone.0007289)

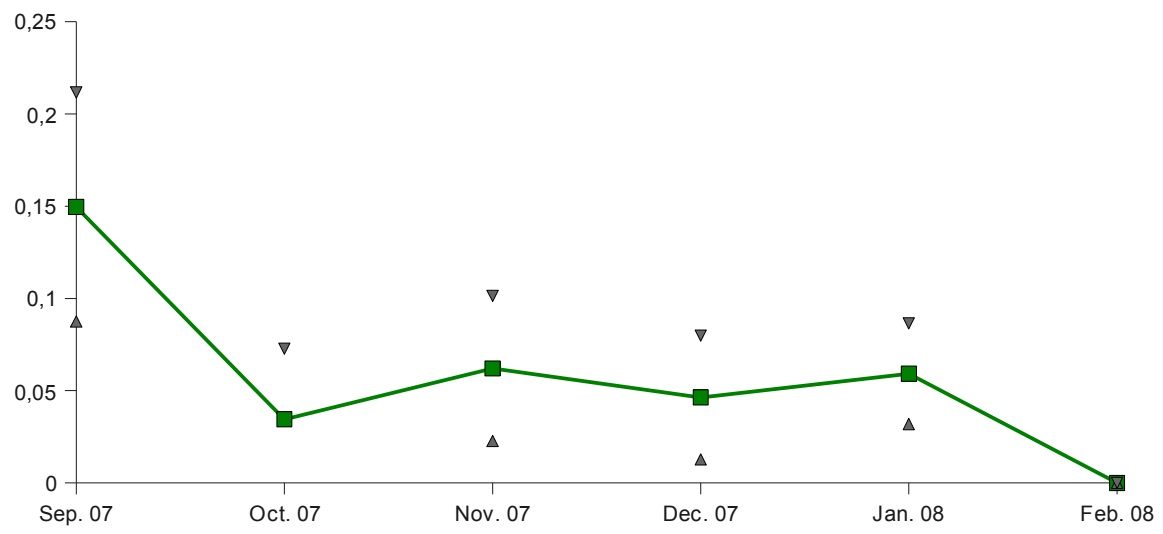

Supplement: Figure S1 — Avian influenza virus prevalence in Common Teal in the Camargue, during winter 2007–2008 (triangles represent 95% confidence interval). (0.02 MB PDF) [file pone.0007289.s001.pdf]

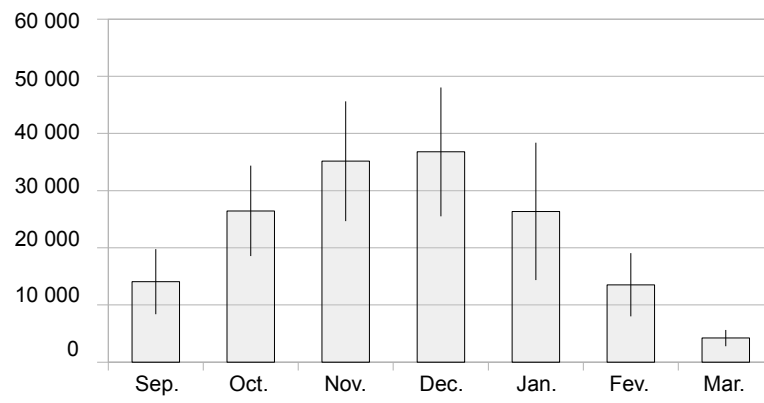

Supplement: Figure S4 — Mean abundance (and standard deviation) of Common Teal in the Camargue, computed between 1964 and 1995. (0.02 MB PDF) [file pone.0007289.s004.pdf]
